# On Apoplexy

**Published:** 1802-06

**Authors:** 


### To the Editors of the Medical and Phyfical Journal.

Gentlemen,

In looking over Dr. Langflow's Remarks upon my Communication, on Apoplexy, in the laft Number of the Medical and Phyfical Journal, my firft intention was to have paffed them over in filence and unnoticed, as they did not feem to me, in any degree, to meet my reafoning and obfervations upon that fubject. I have, however, been induced to forego this intention, judging it more candid and refpectful to Dr. L. to offer a few remarks on the accuracy and propriety of his Statement; and in doing this, the caufe of utility may be ferved, as perhaps fome light may be thrown upon the queftion under confideration, by the acceffion of fome farther obfervations pertaining to the nature and treatment of Apoplexy in general. On this occafion, I fay, I might have remained filent, becaufe it does not appear, that Dr. L. has, in any of his animadverfions, attempted to afcertain the ftate of the brain, in the act of vomiting; neither has he attempted to prove, that the volume of the blood will, in any ftate of the body, more than fill the cavity of the veins, by fhewing the exiftence of plethora ad vafa, or the rupture of veffels from the volume of blood furpaffing in dimenfion the cavities of the veffels. He is alfo filent upon the lofs of vitality, and confequent torpor and inirritability of the brain as favouring Apoplexy, a neceffary effect of the changes of the body as age advances. Neither has any thing been faid againft the obfervation, that it is probable, for the moft part, the effufion of ferum or blood, obfervable in diffecting Apoplectics, may be an effect inftead of a caufe, from the well-known fact of the peculiarity of fuch bodies to evafation of the fluids after death. My reafonings upon thefe fubjects have not been touched upon by Dr. L. and I may, therefore, for the prefent, confider not only them, as a kind of *sarta tecta*, but perhaps fome other parts of my reafoning upon which he has animadverted; fuch as my conjecture, that the caufe of Apoplexy may at times be feated in the lungs, and my doubts about the exiftence of abforbents in the brain.

How far Dr. L. is correct in complaining that I have written under an affumed name, may, I think, with great reafon be doubted; in my arguments and obfervations, I truft, I have preferved the ufual, the neceffary, courtefy and delicacy due from one inquirer, one friend of fcience, to another; and having permitted no injury of character to be offered, no perfonal invective to efcape me, I think this complaint wholly without foundation. On the contrary, Dr. L. needs not be told, that in morals, in politics, in all ſcientific purſuits and literary inquiries, it would be deſirable and uſeful, that opinions ſhould be weighed and eſtimated apart, and without regard to perſons, or the names or characters of their authors; by ſuch proceeding their value and merit would be really and impartially aſcertained, and would be uninfluenced by the paſſions of party; neither would they be aggrandized on the one hand by the warmth of friendſhip, nor depreſſed on the other by the virulence of enmity: I ſhall therefore preſerve my aſſumed name, although it is a matter of real indifference to me, if I am known to Dr. L. by my real name.*

And his charge of affectation, in referring to authors, will be found equally unfounded and unjuſt; for as in writing, it is but common juſtice to refer opinions to their proper authors, ſo quotation will be ever neceſſary, in giving the hiſtory of opinions. But it would ſeem, either that all authors that have not fallen within the limits of Dr. L's reading, or that exiſt not within the pale of his library, are to be conſidered as ancient; or that he has given a latitude to the interpretation of the term ancient, not uſually adopted. According to his interpretation we ſhall have antiquated Seers of yeſterday and Ancients of to-day; for by an unaccountable miſtake, Dr. L. has enumerated among his ancients, Caldani and Lupi, who, if they have eſcaped the ravages of warfare, are Profeſſors ſtill living in Italy. This weakneſs of quotation, and of ancients according to Dr. L. I am fearful I muſt ſtill perſiſt in; and perhaps in the ſequel it will be found, that it would have been uſeful to him, had he indulged a little in the ſame weakneſs.

For a juſtification of what I have ſaid of the facility with which Dr. L. has ſpoken of removing Apoplexy, I need only recall his attention to his own writings, in which he has attempted to ſhew, that in the afternoon he has effected the removal of that fluid from the brain, which had been effuſed in the morning, and by its preſſure had induced a fit of Apoplexy. This facility ſeems at variance with all experience on this ſubject, which tells us, that the fatality of affections of the brain ariſes in a great meaſure from the difficulty of abſorption in that organ. Hence the effects of external injuries, of tumors in the brain, of the effuſion in hydrocephalus, are ſo difficultly reſiſted.

There are ſome other parts of Dr. L's remarks, which I ſhall now proceed to examine more particularly, as they are brought forward in a high tone of confidence, and with the ſemblance of anſwers to my objections and reaſoning.

I ſhall not dwell long upon the ſuppoſed importance of the diſtinction of Apoplexy into ſanguineous and ſerous, Dr. L. having

* The Editors aſſure Dr. L. that Pyrrho is not Dr. Girdleſtone.

having, by his remarks and explanation, given up that diſtinction altogether; for by having admitted, that it is only neceſſary in Apoplexy, to adjuſt the degree of our curative means to the age, the ſtrength, and ſex of the patient, he has admitted there is in Apoplexy nothing but what is common to every other diſeaſe, and therefore the peculiar and ſpecific diſtinction, which he deemed heretofore ſo important, is done away.

On the ſubject of abſorption in the brain, if my judgment deceives me not, it will appear, that by the expreſſion "inhaling viſcus," Dr. L. has either made a great diſcovery in anatomy, or has made uſe of terms without meaning. As far as my reſearches have gone in Anatomy and Phyſiology, I have always underſtood, that the office of abſorption muſt be performed, either by the red veins or the lymphatics; and in later times, it has been ſhewn by accurate and repeated experiments, that the buſineſs of abſorption is the excluſive privilege of the lymphatics; and has been therefore transferred from the veins to them. This being the caſe, and no lymphatics having been proved to exiſt in the brain, I would aſk, what are the organs or inſtruments compoſing the inhaling viſcus? and, how are they called?

I ſhall now proceed to my conjecture, that the cauſe of Apoplexy, may at times be found in the lungs; "a more wild and fanciful hypotheſis than has ever been ſeriouſly promulgated." In tenderneſs to Dr. L. I am almoſt willing to hope, that he does not wiſh any meaning to be affixed to theſe words, but rather, that they ſhould compoſe an expletive ſentence, or be a rhetorical figure, or a kind of *bruta fulmina*; for if this be not the caſe, I muſt think a ſingular fatality attends his efforts in this inquiry, as, unfortunately, among his ancients, I find the cauſe of Apoplexy has been, at times, already referred to the lungs, and by writers, the authority of whom he muſt readily admit; and I hope he will not accuſe me of affectation, when I refer him on this ſubject to Sauvage, and to Prof. Walter* of Berlin, in his Commentary on Apoplexy. Hence it appears, this conjecture, ſo wild and barbarous in Dr. L's opinion, has been long domiciliated and enrolled in the Temple and Tables of Æſculapius. On this ſubject any farther remarks would be idle and petulant.

In my former communication, I attempted to ſhew, that the loſs of vitality, which the brain ſuſtains in common with other parts of the body in the progreſs of life, is the moſt general cauſe of Apoplexy. This loſs pervades the venous ſyſtem

* Vide J. G. Walter, de Apoplexia.

ſyſtem of the brain, and may render it leſs equal to the purpoſes of circulation; and if we reflect, we ſhall find in the ſtructure of the ſinuſſes of the dura mater, and in ſome other peculiarities attending the circulating ſyſtem of the brain, ſufficient reaſons to explain, why, as life advances, an interruption of the circulation ſhould take place there, rather than in any other part. Upon inquiry, it will be found, that from the ſtructure of theſe ſinuſſes, their power as hollow muſcles muſt at all times be feeble, in propelling the blood and promoting the circulation.

And in addition to this ſtructure of the ſinuſſes, it may be obſerved, there are other peculiarities proving that the irritability of the venous ſyſtem of the brain is exerted and expended in a greater ratio than in any other part. From the circumſtance of the veins having no valves; from the motion of the blood in them being unaſſiſted by the neighbourhood of any voluntary muſcles; or by the pulſation of arteries running parallel with them; and alſo by the perpetual diſtenſion of the brain, in the act of expiration.

And if, moreover, it be admitted, that a greater quantity of blood is thrown upon the brain than upon any other equal part, and that from its proximity to the heart, ſuch blood being loaded and charged with oxygen, exerts a more ſtimulant power upon its veſſels than if it were placed at a greater diſtance, a farther reaſon will occur why the irritability or vitality of the veſſels of that organ ſhould be diminiſhed in a greater ratio than in any other part. Thus both the quantity and quality of the blood as ſtimulant, conjoined with the forementioned peculiarities of ſtructure, in the progreſs of life, induce inirritability of the brain and its veſſels, which, when partially ſhewn and expreſſed, gives riſe either to habitual or chronic vertigo, or to palſy, and when more generally, to apoplexy. And upon the ſame reaſoning, it will appear, why the effuſion of ſerum and of blood (but as an effect) ſhould ſo frequently be found, in the diſſection of apoplectics. For from the diminiſhed contractile power or irritability of the veſſels, their fibres will be elongated in every dimenſion, their pores become enlarged, and their ſtrength and denſity diminiſhed as living ſolids, and hence, at firſt congeſtion, and ultimately evaſation of the circulating fluid will take place.

From this reaſoning, it would alſo ſeem, that Apoplexy, in moſt caſes, is the natural termination of life, or a common and neceſſary expreſſion of age, in the ſame manner as takes place in the mortification of the toes of old people.

And in this view of Apoplexy, as ariſing from the defective vitality of the veſſels of the brain, it will appear, that the occurrence occurrence of this difeafe may be antidated, and take place, fooner than would be, from the neceffary and inevitable changes of the body. This earlier occurrence may happen from any caufe unduly affecting the circulation, or diminifhing the ftimulant power of the blood. Thus, in advanced life, much fatigue of body or mind, or errors in diet, by diminifhing the moving powers, may induce Apoplexy. And it may alfo be obferved, that an attack of this difeafe may be favoured, either by the lungs becoming lefs ready conductors of oxygen, by change in their texture, or by the air we breathe not fupplying the wonted portion of oxygen, either from change of feafon, of temperature, or from other caufes affecting the atmofphere. For from experience it would feem, that without oxygen the vafcular fyftem would ceafe to act; and as any defect of this principle would be felt more immediately upon that part of the fyftem where the greateft torpor prevails, it would thence follow, that the circulation in the brain would be more readily affected than in any other part.

But this inirritability of the general fyftem of the brain happening in age, and opening, by Apoplexy, a neceffary and natural avenue to death, muft be cautioufly diftinguifhed from affections of that organ, which may happen at all periods of life, as from hæmorrhage by external accident, or from the difeafed ftate of the coats of a veffel or veffels, fuch affection being a mere local difeafe.

And this opinion, that, if life were not prematurely terminated by the intervention of accidental difeafes, from contagion, intemperance, or other means, and if the living powers had their play unimpeded, and the preordained or neceffary change from life to death proceeded in the moft regular and natural manner, it is probable, that the approach of death would take place either by Apoplexy interrupting the circulation of the brain, from the diminifhed irritability of its veffels, as above explained, or by gangrene in the extremities, which being placed the moft diftant from the heart, the centre of motion, enjoy a lefs active vitality, and of courfe would fooneft, by lofs of circulation, undergo the change from life to death; is greatly confirmed by the fentiments of Profeffor Walter, who has expreffed himfelf on this fubject in the following manner. "Plurimi hominum diuturnam fibi optant vitam; fi ideo tales decrepitos inveftigamus, videbimus omnes homines ætatem fenilem adfequutos aut Apoplexia aut gangrænofa inflammatione remotiffimarum partium corporis, e. g. manuum vel pedum, mori. Si itaque comparemus modum mortis hominum fummæ fenectutis, qui vel Apoplexia vel inflammatione fphacelofa perierunt, tum experientia docet, ex decem talibus decrepitis certè

certè novem Apoplexia, decimum verò inflammatione gangrænosa mori. Hæc contemplatio nobis jus tribuit dicendi, modum quo in senectute morimur, planè simplicem esse."

In this view of Apoplexy as a necessary cause of death, or as the result of the changes necessarily incident to the body from age, a reason will occur why its effects are so rarely wholly removed; and from this view, Dr. L. may, perhaps, experience some feelings of regret, when he reflects upon the intemperate, the undiscriminating intrepidity of conclusion which he has exercised, and upon the zeal and confidence with which he has spoken upon this subject; a mode of conduct equally unworthy a liberal and judicious Inquirer.

This theory, it must be confessed, is humiliating, as it places the means of relief and indications of cure, in much doubt and uncertainty, by making this disease, frequently, a mere expression of waning or departing life; but such as it is, I submit it to your readers, and, desiring that it may be received with doubt, and examined with caution, I shall conclude, in the spirit of the Elian Philosopher, whose name I have assumed, although with the words of a Christian Father, by saying, " Magis eligo cautam ignorantiam confiteri, quàm falsam scientiam profiteri."

PYRRHO.

